# Factors Which Facilitate or Impede Interpersonal Interactions and Relationships after Spinal Cord Injury: A Scoping Review with Suggestions for Rehabilitation

**DOI:** 10.1155/2016/9373786

**Published:** 2016-12-29

**Authors:** Delena Amsters, Sarita Schuurs, Kiley Pershouse, Bettina Power, Yvonne Harestad, Melissa Kendall, Pim Kuipers

**Affiliations:** ^1^Spinal Outreach Team, Metro South Health, P.O. Box 6053, Buranda, QLD 4102, Australia; ^2^Transitional Rehabilitation Program, Metro South Health and Menzies Health Institute Queensland, Griffith University, Meadowbrook, QLD 4131, Australia; ^3^Centre for Functioning & Health Research, Metro South Health and Menzies Health Institute Queensland, Griffith University, Meadowbrook, QLD 4131, Australia

## Abstract

Interpersonal interactions and relationships can influence an individual's perceptions of health and quality of life in the presence of disability. In the case of people with spinal cord injury (SCI), positive interpersonal interactions and relationships have been shown to contribute to resilience and adaptability. Understanding factors which facilitate or impede the development and maintenance of relationships after SCI may form the basis for proactive relationship support for people with SCI. To gain a broad insight into these factors, a scoping review was undertaken. Databases were searched for English language studies published between 2000 and 2015 that informed the review question. Sixty-two (62) studies were identified. Thematic analysis was conducted on data extracted from the studies and 51 factors which may facilitate relationships and 38 factors which may impede relationships after SCI were noted. The majority of factors could be categorized as environmental or personal according to the domains of the International Classification of Functioning, Disability, and Health (ICF). The facilitating factors included partner and social support, reciprocity in relationships, and presenting oneself positively. Impeding factors included physical environmental barriers, real and perceived social biases, and poor self-image. Factors identified may inform the provision of supportive, holistic rehabilitation for people with SCI.

## 1. Introduction

Most people experience many different types of relationships in their lives. These range from acquaintanceship, which may be formal or informal, friendships, kinship bonds, and romantic or intimate relationships [1]. Friendships, which are usually thought of as voluntary relationships, are generally underpinned by affection, companionship, trust, and reciprocity [2]. Positive intimate partnerships are characterized by commitment and closeness and may change and evolve based on partners' efforts at relationship maintenance [3]. The importance and meaning of sexuality and sexual intimacy within such relationships will vary from couple to couple; however evidence suggests that it remains an important component of such relationships in the long term and in the presence of factors which may be barriers to sexual intimacy [4]. Kinship or family relationships are influenced by social and cultural norms but less so in Western countries where families are becoming less standardized in composition. Consequently, roles and responsibilities within family relationships are more fluid and flexible [5]. Regardless of the personnel who make up a family, they may provide practical and emotional support for one another [6]. The World Health Organization has recognized the importance of “interpersonal interactions and relationships” as a domain of human functioning, by including it as a chapter in the International Classification of Functioning, Disability, and Health (ICF) [7].

Factors which might adversely affect the development and maintenance of interpersonal interactions and relationships include the presence of disease conditions [8], physical, psychological, or cognitive impairment [9], and environmental barriers to social participation [10]. In the case of spinal cord Injury (SCI), the most obvious consequence which may restrict an individual's capacity to participate socially is a mobility impairment which can be exacerbated by aspects of the natural and built environment [10, 11]. There are, however, other hidden sequelae, such as bladder, bowel, and sexual dysfunction, and secondary health conditions, such as persistent pain and pressure injury, which can have a negative impact upon social participation [10, 12–14] including relationship formation and preservation.

Research in the USA, conducted in the 1980s, found lower marriage rates after SCI and higher divorce rates for preinjury marriages when compared with the general population [15]. The financial cost of SCI and physical and psychological burden on spouses, as well as the changed nature of relationship roles, have been suggested as sources of relationship strain in the presence of SCI [16]. Conversely, some studies have reported richer, more meaningful interpersonal relationships as an outcome of SCI [17–19]. While relationships may be negatively affected by the presence of disability, healthy, robust relationships have the capacity to positively impact individuals' perceptions of health and quality of life in the presence of disability [20–22]. For example, positive spousal relationships have been associated with reduction in functional limitations and depressive symptoms for people with vision impairments [23] and less disability for women coping with persistent pain [24]. Similarly, partner support has been shown to have a positive effect on self-esteem and well-being for people with multiple sclerosis, particularly when they reciprocate the support [25]. For people with SCI, the quality of relationships with family and friends has been identified as contributing to resilience and adaptability [26]. Social support has also been linked to positive psychological outcomes for people with SCI [27].

It is evident that SCI has the potential to disrupt the development and maintenance of interpersonal relationships. It is also apparent that social and personal relationships after SCI can positively influence outcomes for the individual with SCI. It is logical, then, that understanding the factors which facilitate or impede relationships after SCI will form the basis for more proactive relationship support.

The purpose of this review was to examine the literature for evidence of factors which facilitate or impede the development and maintenance of interpersonal interactions and relationships after SCI. The outcome of the review will be used to identify key areas worthy of closer scrutiny and possible incorporation into rehabilitation interventions.

## 2. Method

Rumrill et al. [28] described scoping reviews as “efficient ways of identifying themes and trends” in a topic area which is broad in nature and incorporates studies of differing approaches. Scoping reviews can cover a breadth of literature and draw information from qualitative and quantitative studies and typically do not include formal evaluations of the quality of the included research. The characteristics of a scoping review made it a suitable approach for exploration of factors facilitating or impeding interpersonal interactions and relationships after SCI. This scoping review was conducted with reference to the methodological framework described by Arksey and O'Malley [29]. A thematic analysis of the studies was conducted to produce a basic synthesis [30].

The research question to which the scoping review was addressed was “what factors facilitate or impede the maintenance and development of interpersonal interactions and relationships for people who have SCI?”

### 2.1. Search Procedure

Search terms for interpersonal interactions and relationships were extracted from Chapter 7 of the ICF (see Table 1). These terms seek to cover the breadth of the topic which ranges from general day-to-day interpersonal interactions to personal interactions with particular people, including formal relationships, informal social relationships, family relationships, and intimate relationships. Each term was searched in conjunction with “spinal cord injury/injuries” in the databases Cinahl, Medline, Psych Info, and Psychology and Behavioural Sciences Collection. The search was limited to peer-reviewed articles published in English and relating to human studies between 2000 and 2015.

Studies were considered for inclusion if the participant group included (but was not necessarily exclusively) adults with SCI. One study examining the dating experiences of adolescents with SCI was found and retained as it explored concepts relevant to adult relationships. Studies which primarily examined factors influencing social participation or social integration were included if it was evident that interpersonal interactions and relationships were a component of the study. Studies were excluded if they did not identify one or more factors which facilitate or impede interpersonal interactions and relationships. Studies which only examined the viewpoints of relatives, friends, or caregivers were also excluded from this review.

**Figure 1:**
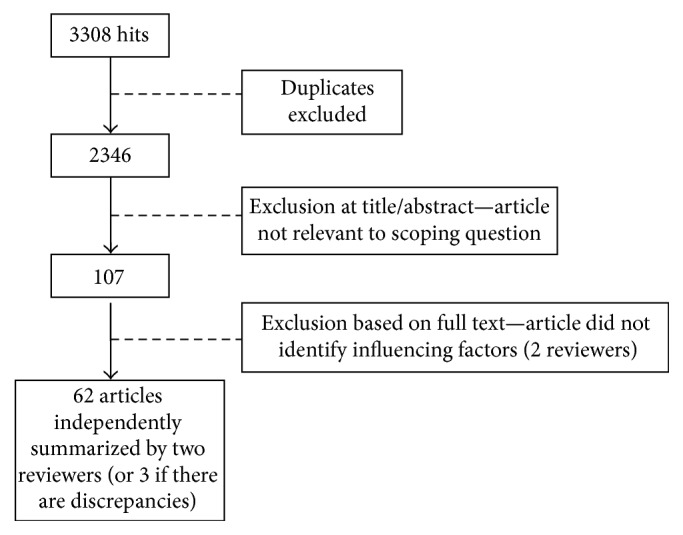
Flowchart of study selection process.

Figure 1 shows the study selection process. Sixty-two (62) studies were ultimately selected and there were no additions from reference list searching. Data from the retained studies were independently extracted by two members of the review group by following a data extraction template. The template included study purpose; primary construct examined by the research; context in which the research was conducted; description of the participants; methodological approach; and factors identified which facilitate or impede interpersonal interactions and relationships after SCI. The two extracted summaries prepared for each study were then compared, and a third reviewer was asked to examine the study in cases where the summaries were discrepant.

### 2.2. Analysis

The characteristics of the studies reviewed were summarized descriptively with respect to countries in which the studies were set, the primary construct of the studies, and the methodological approach of each study. The participant groups were summarized by gender and whether they were mixed groups or exclusively people with SCI.

All factors which were identified as influencing interpersonal interactions and relationships were subjected to statement-by-statement coding for thematic content. Only statements which clearly indicated a causal effect on relationships, grounded in the study data, were coded. The code label represents the causal agent; that is, “how you feel about your injured body affects how you are with people” would be coded as “body image.” This approach to coding was based on the semantic approach described by Braun and Clarke [31]. Primary coding was conducted by one reviewer (DA) with the aid of NVivo 8™ software. Each factor statement was also coded as either* facilitating* or* impeding* with respect to the direction of its influence on interpersonal interactions and relationships. Codes were grouped based on the domains of the ICF—body structure/function factors (i.e., changes to physiological functions and anatomical components of the body); activities factors (i.e., execution of tasks or actions); participation factors (i.e., involvement in life situations); environment factors (i.e., equipment and technology, physical environment, support, attitudes, and service systems); and personal factors (e.g., gender, age, coping styles, education, and behaviour patterns). The ICF provides a ready-made framework for organizing and interpreting coded data as it gives consideration to the major components of human functioning in the presence of a health condition—in this case interpersonal relationships in the presence of SCI [7]. The environmental and personal factors were by far the largest group of codes and so were subsequently grouped at a secondary level. The secondary groupings were factors pertaining to social attitudes, supporters, physical environment, and resource factors (which broadly encompassed equipment and service systems). The personal factors were further grouped into either fixed or modifiable factors.

The primary codes, direction of influence, and ICF groupings were reviewed by two other reviewers (MK and PK) to confirm accuracy of coding and conceptual clarity. Minimal amendment was required to reach agreement between reviewers as to the accuracy and clarity of codes. This involved creating a “peer support” code within the environment (supporter) factors and moving “attachment style” to the fixed personal factors, as well as some changes to coding nomenclature.

## 3. Results

Table 2 shows the characteristics of the literature included in the scoping review. The majority of studies were set in either the USA (26) or Canada (13). The primary construct examined in the papers was spread across 15 topics with “sexual relationships and sexuality” and “personal relationships” being the two most frequent. Thirty-four (34) studies had a qualitative design, 25 were quantitative, and three (3) utilized mixed methods. Eleven (11) studies had only women participating and eight (8) had only men. The gender breakdown was not clear in five (5) studies reviewed. Four (4) studies involved participants with a variety of disabilities and six (6) studies incorporated comparison groups of family members or control participants.

Coding of factor statements yielded 89 codes—51 factors which facilitate relationships and 38 factors which impede relationships.  Table 3 shows the codes grouped into the ICF domains. For each code, the studies which contained the supporting data are shown.

### 3.1. Body Structure and Function Factors

The most frequently cited* body structure and function* factors which impeded interpersonal interactions and relationships pertain to bladder and bowel dysfunction [42, 54, 55, 57, 60, 75, 82, 90]. As well as suffering the actual embarrassment of incontinence (particularly during intimate moments), there was a fear of incontinence which held some back from fully engaging with intimate partners and in other social situations. One study found that Sildenafil was viewed positively for its role in enhancing sexual intimacy [38].

Four (4) studies suggested that those with greater impairment faced particular impediments to socializing and forming relationships [32, 42, 58, 71] and, conversely, that those who had a lesser degree of impairment had better social participation and relationship outcomes [37].

An individual's state of health has been suggested to influence social participation and relationships for people with SCI, both positively [47, 58, 63, 72] and negatively [58]. Secondary impairments to body structure and function from SCI, including pressure injuries and pain, can be socially limiting, while general physical well-being will maximize social opportunities.

### 3.2. Activities Factors

The time-consuming nature of carrying out activities of daily living, dependence on others for personal care, such as bladder management, and the inability to act spontaneously were all cited as impeding socializing, forming relationships, and enjoying sexual intimacy [34, 55, 59, 63, 66, 81, 90]. Independence can positively influence the experience of sexuality [43] and higher levels of mobility can impact couple satisfaction and cohesion [83]. Interestingly, results from the study by Bastanfar and Crewe [33] suggested that using a wheelchair for mobility might make a person stand out in social situations. This could be an advantage for meeting new people but might be construed by some as an obstacle to deeper intimacy [33]. Ability to maintain continence and be independent in bladder management were positive social and relationship factors [43, 71].

### 3.3. Participation Factors

Meeting people and developing social networks through recreation and sporting activities [34, 36, 37, 40, 61, 86] as well as work place interactions [50, 51, 76, 89] were frequently cited as facilitators. Unemployment was associated with poorer relationship outcomes [32, 47, 66].

### 3.4. Environment Factors

#### 3.4.1. Social Environment Factors

Some studies highlighted the role that social environments could play in positive interpersonal interactions and relationships after SCI. Clubs, businesses, and organizations that created social settings were acknowledged [58, 66]. One study mentioned the value of social settings which facilitated the sharing of experiences with others with SCI [74].

The role that rehabilitation programs could play in promoting social environments was an interesting finding of some studies. It was suggested in one study that therapists can promote social participation by acting as social partners for people with SCI in the early stages after injury [52]. Rehabilitation programs which are inclusive of friends and family have also been said to maintain and strengthen relationship bonds [17, 52, 68]. Rehabilitation which includes increasing exposure to social settings for people with SCI, with an opportunity for debriefing after such experiences, was presented positively [59].

Specific programs designed to enhance interpersonal interactions and relationships were examined in two studies, with some limited evidence of success [44, 85]. One of these programs approached this directly through psychoeducation aimed at assisting with dating and relationships [44] whilst the other was an outdoor sports program which anticipated that friendships and social support would be created as a by-product of the program environment [85]. Vocational rehabilitation programs were also implied to improve social integration and relationships in two studies [51, 89]. One study suggested that people with high level SCI who had experienced institutional living arrangements were very socially restricted by such environments [68]. Another study stated that lack of privacy in rehabilitation was an impediment to “feeling attractive, social and interested in intimacy” [80].

Nine (9) studies suggested that social bias hampered people's ability to make new relationships after SCI [18, 33, 41, 42, 63, 80, 81, 84]. This social bias could be tangible, such as external pressure from family members against getting involved with someone with SCI, or it could be a perception based on the internalized beliefs of the person with SCI. In two studies, it was mentioned by participants that they just had not met the “right” person who would be willing to enter into a relationship [75, 81]. One study reported that cases where the SCI was caused by violence were associated with separation and divorce which may be linked to broader social disadvantage [32].

#### 3.4.2. Resource Factors

Enabling equipment [58], assistive technology [66, 87], and, in particular, communication technology [45, 53, 56, 64, 66, 81, 87] were referred to as important facilitators of interpersonal interactions and relationships. Lack of a mobile (cell) phone was raised by one study as a significant negative factor for interpersonal interactions [53]. Those without a phone had less contact over the course of a month with friends, business contacts, and strangers.

Access to quality information and education resources, particularly with respect to sex and sexuality after SCI, were seen as important [42, 49, 80–82]. The information and education were seen as needed to allay fears and promote openness between partners. Song [88] found informational support to have a positive effect on social integration but the exact nature of this information was not specified. Two studies mentioned a negative impact on social and personal relationships due to transport problems [58, 71]. Having a service dog was found in one study to be a facilitator of social interaction [62].

Financial pressure was associated with stress on relationships [34, 82] and financial resources influenced the ability to participate in social activities [39, 58]. The presence or absence of insurance was also mentioned in three studies as a financial factor impacting upon relationships [37, 66, 82].

#### 3.4.3. Supporter Factors

Factors which were suggested to consolidate partner relationships after SCI included understanding, acceptance, and support from the non-SCI partner [16, 34, 72, 80–82]; the presence of children, which leads to shared focus and purpose [34]; and spending quality time together and with family [34, 41]. The degree of social integration of persons with SCI was also associated with persistence of partner relationships over time [47].

Support from partner, peers, friends, and family was found to be associated with increased social participation for people with SCI [58, 59, 66, 74]. In a virtuous cycle, it is the quality of the support network that creates the opportunity for people with SCI to reestablish aspects of their identity and thus strengthen relationships [17, 72, 78, 81].

The facilitating effect on relationships of reciprocity and mutuality was the most commonly coded supporter factor [16, 17, 46, 52, 68, 77–79]. The reciprocal nature of relationships was recognized as vital, even in the presence of catastrophic SCI. For couples (where one person has SCI) this reciprocity may mean supporting one another to cope and doing activities together.

When a partner or family member takes on caregiver duties for the person with SCI, this has been suggested as potentially deleterious to some relationships [41, 82]. Access to instrumental support from external sources may act as a buffer against this effect [79]. Studies also suggested that some partners or friends simply cannot cope with the changes associated with maintaining a relationship with a person with SCI and the negativity of partners or friends could make relationships untenable [18, 65, 67, 73, 82]. In trying to establish intimate partner relationships after SCI, it may be the objections of family and peers which undermine the relationship [82, 84].

With respect to sexual intimacy, being married was found in one study to be associated with decreased sexual satisfaction, though “sentimental life” was found to be better for married couples [71]. Other aspects of life, such as caring for children, may influence availability and willingness to engage in sexual intimacy in the presence of SCI [42].

There was some evidence to suggest that partner relationships established after injury are likely to be more successful than preinjury relationships [49]. Where relationship problems existed before SCI, these may be magnified after SCI [49]. A strong preinjury relationship may be protective after SCI [16], and people who do not experience changes in partner relationships after SCI tend to be more satisfied with their intimate lives [69]. The passage of time (over a five-year period) was associated with increased separation and divorce rates in one study [32].

#### 3.4.4. Physical Environment Factors

Eight (8) studies highlighted aspects of the physical environment, both natural and built, that could have negative consequences for social activity and development of relationships. Physical environmental challenges such as uneven terrain and adverse climatic conditions may reduce the likelihood of social activities outside the home [66]. Similarly, inaccessibility of the built environment including friends' homes and community social venues was reported as challenging in establishing and sustaining relationships [52, 58, 63, 66, 67, 82]. Concern about accessible toilet facilities could also limit choices for social participation [55].

One study reported interesting positive findings with respect to the physical environment and social integration and participation. Botticello et al. [35] found that social participation was enhanced in communities that had open spaces. Conversely, communities with greater residential density lowered the odds of social participation for people with SCI. This was cautiously interpreted as potentially relating to factors such as the positive aesthetic of open spaces and possible access and safety issues in residentially dense areas.

### 3.5. Personal Factors

#### 3.5.1. Fixed Factors

A number of personal factors that are fixed (or are not readily changed) were identified as potentially influencing interpersonal interactions and relationships. Level of educational attainment [39, 47, 54] and cognitive scores [37] have been suggested to influence social participation and relationships for people with SCI. Relationship outcomes after SCI have been found to be influenced by age. Being younger after SCI has been associated with better sexual life [71] and is also a positive predictor of social integration [37]. Conversely, being older with SCI was associated with negative impacts on sexual relationships [42] and on interpersonal interactions [39, 70]. However, being younger was associated with higher likelihood of separation and divorce [32] and being older was associated with marriages surviving longer [47].

Two studies examined differences in social outcomes after SCI for particular ethnic groups in the USA. Shorter time to divorce [47] and lower levels of social integration [37] were associated with being African American versus Caucasian. Chan [16] suggested that cultural traditions may facilitate the maintenance of relationship in the presence of SCI, such as cultural traditions mitigating divorce.

Gender has been suggested to have a bearing on interpersonal interactions and relationships after SCI. Women, more so than men, may work to maintain contacts with family and friends [39]. Satisfaction with sexual life after SCI has also been reported to differ by gender, with men being less likely to be satisfied than women [71].

Attachment styles displayed by adults are understood to affect the development and maintenance of romantic relationships. A study by Hwang et al. [46] examined the attachment style of participants with SCI and found that secure attachment style was associated with more satisfying relationships. Avoidance (one dimension of attachment style) was strongly inversely related to couple satisfaction, couple consensus, affective expression, and total dyadic adjustment. Anxiety (the second dimension of attachment style) was inversely related to dyadic cohesion. Results were consistent with non-SCI populations.

#### 3.5.2. Modifiable Factors

The thoughts, feelings, and resultant behaviours of individuals with SCI may affect their chances of forming new relationships. Thoughts and feelings such as fear of rejection [82], retaining bitterness after breakups [49], and withdrawing due to social discomfort [59] have been suggested to reduce the chances of positive relationship outcomes after SCI. Behaviours such as failing to be “up front” and open about disability [81], being passive rather than proactive in seeking new relationships [33], and “opting out” of partner interactions, possibly in favour of other life roles [49], have all been shown to have negative consequences for social interactions and relationships after SCI. On the other hand, making an effort [77], being proactive [52, 67, 74], putting effort into self-presentation [52, 59, 80–82], and accepting help when it is needed [65] were said to facilitate development of relationships. Exhibiting patience and acceptance [16] and placing value on relationships [16, 34, 52, 68, 82] were also suggested as behaviours which may help to maintain relationships in the presence of SCI.

Open communication was frequently mentioned as positively associated with good relationship outcomes [16, 49, 77, 80, 82]. Good interpersonal skills before injury (including good communication skills) persist after injury. Those who were good at meeting people and “romancing” before SCI are those who are more successful after injury [33]. It was suggested in one study that an individual's social skills need to be of a high level to successfully “navigate” the social world after SCI [52].

The most frequently coded factor amongst the modifiable personal factors was poor self-image. Perceiving oneself as physically unattractive [42, 49, 75], having a poor sexual self-image [80–82], and considering oneself as unfit for a relationship due to disability [49, 59, 81, 84] have been shown to reduce the chances of achieving positive social and personal relationships. Having a decreased interest in sexual intimacy [75] was also cited as potentially deleterious to relationships. Conversely, broadening one's personal definition of sexuality [42, 49, 80–83] and having a positive self-image [36, 42, 52, 81] were associated with social and relationship success. The passage of time may allow the reconstruction of an individual's sexual identity [81] and the opportunity to have positive sexual experiences may also play a role in this reconstruction [42].

Interestingly, certain cognitive schemata (relating to giving and receiving support within couple relationships) were found to be more prevalent in a sample of men with SCI when compared with nondisabled control participants [79]. These schemata related to schema of support, availability of support, and supportive behaviours. There is an assumption drawn that support schemata shape support behaviour, and therefore helpful cognitive support schemata are assumed to have an association with positive partner relationships [79]. The ability to socially “self-monitor” is another metacognitive skill that has been suggested to facilitate social outcomes for people with SCI. High self-monitoring by persons with SCI has been shown to be associated with more frequent socializing with friends and going out for fun and relaxation [48].

One study suggested that adhering to faith based norms could be a factor in keeping marriages together in the face of relationship challenges created by the onset of SCI [16].

## 4. Discussion

This scoping review was conducted to examine the literature for evidence of factors which facilitate or impede the development and maintenance of interpersonal interactions and relationships after SCI and to identify key areas worthy of closer scrutiny and possible incorporation into rehabilitation interventions. The majority of relevant English language literature emanated from the USA and Canada. However, in defence of generalizing the findings, it is noted that most of the factors identified do not appear to be linked to the context of a particular country. The exceptions to this may be the influence of climatic conditions, the financial influence of insurance schemes, and factors related to ethnicity and culture.

The greater number of qualitative studies versus quantitative studies reflects the role of qualitative methodologies in revealing the lived experience of SCI, of which interpersonal interactions and relationships are an integral part. The vast majority of the quantitative studies were correlational or comparative in nature, examining associations between variables within and between groups. There was only one study which described and evaluated a specific intervention aimed at equipping people socially after SCI—a dating and relationships psychoeducational group [44]. While the relative lack of such studies does not mean that these types of interventions do not occur in practice, it does suggest a gap in the research to date.

### 4.1. Understanding Functional Facilitators and Impediments

The factors which facilitate or impede interpersonal interactions and relationships after SCI, as extracted from the studies in the scoping review, align closely with the domains of the ICF [7]. The functional domains of the ICF—body structure and function, activities, and participation—are recognized as being influenced by contextual factors. These three levels of human functioning are also interactive with one another and there were a number of examples identified by the scoping review which showed how the functional domains of the ICF were interrelated with participating in interpersonal interactions and relationships after SCI.

Relationship and social problems linked to bladder and bowel function and management were prevalent factors identified in the review. Fear of incontinence is likely to be just as socially limiting as actual episodes of incontinence. Bladder, bowel, and sexual dysfunction are often described as hidden disabilities but have profound impacts on quality of life [91]. It has been suggested that health professionals will best assist people to manage these problems if they understand each individual's circumstances and experiences [57, 92]. Timely access to quality information and education was noted in the review, particularly in relation to sexuality and intimate partner relationships. No single information source will be suitable for all. For some, access to an appropriate health professional will have value, and for others the Internet will provide the answers, while some may prefer talking to peers with SCI.

Strategies for maximizing autonomy in the presence of a high level of impairment may reduce the impacts of physical dependence upon relationships. These include learning from others with SCI, being informed, setting goals, being assertive, planning and organizing, asking for and accepting help, and learning to deal with the reactions of others [93]. Unfortunately, some of these strategies may involve a “trade-off” against spontaneity. The alternative is adopting an approach of “taking life as it comes” [93]. Access to assistive technology and specialized equipment and external support can also help people with SCI to redefine their roles within family relationships, from one of care recipient to partner or parent. Other tangible resources which were noted to assist relationships included access to transportation and use of service dogs. Financial security may underpin access to all such material resources.

Relationship development within rehabilitation programs has often been restricted to providing structure for people with SCI to participate in sport, recreation, and work. While it is clear from this review that participation in these pursuits does provide opportunities for meaningful interpersonal interactions, they have tended to be a by-product rather than the main focus of the intervention. This review, however, has highlighted the strong representation of factors in domains other than* participation* and therefore underscored that it is in these other areas that the most scope for new rehabilitation interventions aimed at relationship development and maintenance may lie.

### 4.2. Understanding Contextual Facilitators and Impediments

According to the ICF, the contextual factors which will influence the individual's experience of disability are both environmental (social, physical, resource, and support) and personal (fixed and modifiable). It was these contextual factors which had the greatest representation in the results of this scoping review.

The role that a supportive partner can play in helping a person with SCI to reconstruct their sexual identity was a prevalent theme in the studies reviewed. Partners were also shown to play a role in supporting the person with SCI to reconstruct their social identity. Successful relationships in the presence of SCI were characterized in this literature as mutual and reciprocal. The notion of “doing together” was highlighted in a number of studies. This may occur through activities that involve shared focus and purpose such as the raising of children. Similarly, it was suggested that people with SCI need to feel they are actively contributing to friendships and that their friends “can count on them.” The benefit of reciprocity and mutuality for successful relationships is clearly not peculiar to SCI [94], but it may be something that is downplayed in the aftermath of a major traumatic event and catastrophic physical loss. In rehabilitation settings, health professionals can provide a supportive framework for people with SCI and their family and friends to reestablish reciprocity and mutuality [95].

Social bias or stigma was cited as an impediment to relationship formation in several studies. Societal pressures, such as a parent not approving of a person with SCI as a partner for their child, may pose a tangible barrier which negatively impacts relationships. However, the way these perceptions are anticipated or internalized by people with SCI may be even more damaging to relationship formation. Cognitive strategies may be of help in building confidence and resilience in the face of real or perceived social bias [96, 97]. Ideally, rehabilitation programs should provide a socially supportive environment which includes opportunities to experience social interaction and time for debriefing with professionals or peers after the experience.

Many of the personal factors identified as influencing interpersonal interactions and relationships are fixed, such as gender, age, cultural background, ethnicity, educational attainment, and cognitive ability. However, many of the behavioural and psychological factors identified may be modifiable through psychosocial intervention. The communication skills and social competence of an individual with SCI are personal factors which have the capacity for support and enhancement [98, 99]. Encouraging and supporting people with SCI to be open, proactive, and positive in social settings could also be helpful in allaying some of the discomfort and anxiety associated with poor self-image. Putting effort into self-presentation was highlighted in some studies as beneficial and is a strategy which is readily supported. In addition, a willingness to accept help, making an effort, and exhibiting patience and acceptance were all suggested as being socially advantageous behaviours in the presence of SCI which can be readily encouraged.

The results in the study by Hwang et al. [46] on attachment styles and the work by Gilad and Lavee [79] on cognitive support schemas may be indicative that much of the research on relationship development and maintenance as it relates to the general population will also hold true for people with SCI. The salient lesson may be that relationship fundamentals do not change in the presence of SCI. For example, engaging in processes that promote mutuality are regular features of healthy couple relationships [100]. If too much emphasis is placed upon the needs of an individual with SCI at the expense of their family and friends or if they are excused or absolved from certain behaviours simply because they have an SCI, this may contribute to relationship deterioration. Educating both the person with SCI and their significant others about these relationship reinforcing behaviours may be helpful.

### 4.3. Limitations of the Scoping Review

This scoping review was exploratory in nature and, in the absence of quality appraisals of the studies under review, the results must be viewed with appropriate caution. Several factors which have been suggested by the review to facilitate or impede interpersonal interactions and relationships were only cited in one study; however all coded factors have been presented for completeness. In the analysis and presentation of results, the factors have been treated simply as stand-alone variables. No attempt has been made to look at either how primary factors may influence secondary factors or how groups of factors may work together to influence relationship outcomes.

The scoping review has touched on a number of areas which intersect with interpersonal interactions and relationships after SCI but is not a comprehensive review of these related topics. Studies examining social participation and social integration were included in the review if the study made reference to relationships or if a measurement instrument was used which included relationships as a domain. It should, however, be recognized that social participation and social integration are concepts that are much broader than relationships alone. This means that some of the factors identified may relate to participation outcomes that reach beyond interpersonal interactions and relationships. The review included a considerable number of studies which primarily focused on sex and sexuality. Many of these had significant content about relationships but those that did not were dropped from the review. It should be made clear, therefore, that the review is not a comprehensive review of sexuality and SCI.

Eleven studies involved only women as participants and this may have biased the results of the review. The number of studies involving women is surprising, as the proportion of men versus women sustaining SCI has been reported at 3.8 : 1 worldwide [101]. It may reflect a recent response to historical underrepresentation of women's issues in SCI research. A significant number of studies which involved sport and recreation were examined in this review and this may be because more men experience SCI, and men are more likely to establish relationships based on shared activities [1]. The data generated from this scoping review were not analysed at a level which incorporated gender as a variable; consequently it is recommended that future studies and reviews should focus on gender based differences in the experience of SCI and in relationship formation in particular. There were also some studies included which gathered data from mixed diagnostic groups. As far as was possible, results which did not relate to SCI were not extracted from the studies but it is possible that the mixed groups may have created some distortion.

In coding and categorizing factors which facilitate or impede relationships after SCI, there is a risk of appearing to diminish experiences which may be unique, personal, and profound to the level of a functional interaction. However, the intent of identifying these factors is to inform the provision of supportive, holistic rehabilitation for people with SCI.

### 4.4. Implications for Future Research and Practice

This review has highlighted that minimal research has been conducted to examine the factors which facilitate or impede relationships after SCI. The review has relied heavily upon incidental findings relevant to relationships or studies which made reference to relationships as part of an investigation into other related constructs. This suggests a need to conduct further research which has a specific focus on the facilitators and impediments to relationships after SCI. Targeted systematic reviews which focus on key factors identified in this review would also further inform the development of rehabilitation strategies.

From the outcomes of the current review, a number of ways that the rehabilitation process might support the maintenance and development of interpersonal relationships after SCI can be inferred. An understanding on the part of rehabilitation practitioners of the impacts of certain factors on relationships, in the presence of SCI, is a logical starting point. For example, episodes of incontinence and fear of such episodes may directly impact an individual's willingness to engage socially and intimately. Rehabilitation practitioners can provide practical strategies as well as psychological support in this regard. Interventions that support the development and presentation of a positive self-image may enhance confidence in establishing and maintaining relationships.

Creating rehabilitation environments which encourage and support social interaction between the person with SCI and family and friends is vital. This might be achieved through creation of shared social spaces, time for social interaction, and flexibility of rehabilitation programing. Importantly, rehabilitation space needs to provide a sense of welcome to these significant others. The review has touched upon interesting information about the role of health professionals as proxies for early socialization after SCI. This is an aspect of rehabilitation which would be interesting to research further in the context of professional role boundaries.

This scoping review, which was confined to peer-reviewed literature, has found limited evidence of intervention programs directly aimed at assisting people to form and maintain relationships in the presence of SCI. However, a review of grey literature may reveal that such programs do exist. Mainstream relationship programs and resources are likely to be applicable in SCI rehabilitation. There may, however, be additions or emphases which enhance the effectiveness of such programs and resources in the context of SCI. Future research could examine the efficacy of various combinations of relationship programs and resources for people with SCI.

## 5. Conclusion

This scoping review has identified an array of factors and highlighted a number of key factors which may potentially facilitate or impede interpersonal interactions and relationships after SCI. Some of these factors may be the focus of targeted support within rehabilitation programs. This may be through formal inclusion in patient education programs, as a focus of one-to-one counselling or through the provision of information resources. There is also evidence from the review to suggest that the inclusion of friends and family in the rehabilitation process is an important part of optimising relationship outcomes after SCI. The concept of social rehabilitation, where people newly injured can be supported to reestablish their social identity, has also been raised by this review. A more targeted systematic review which focuses on key factors identified in this review would be the next step towards informing the development of rehabilitation strategies.

## Figures and Tables

**Table 1:** Search terms for interpersonal interactions and relationships.

interpersonal	child^*∗*^
relationship^*∗*^	parent^*∗*^
social^*∗*^	sibling^*∗*^
friend^*∗*^	spous^*∗*^
neighbour^*∗*^	partner^*∗*^
acquaintance^*∗*^	roman^*∗*^
peer^*∗*^	intima^*∗*^
family	sexual^*∗*^

The asterisks are meant to indicate truncation.

**Table 2:** Characteristics of the literature included in the scoping review.

Setting	Studies
USA	[17, 18, 32–55]
Canada	[56–68]
Italy	[69–72]
Scandinavian countries	[73–76]
Israel	[77–79]
Australia	[80, 81]
Hong Kong	[16, 82]
South Africa	[83, 84]
UK	[85, 86]
Ireland	[87]
Korea	[88]
New Zealand	[89]
Turkey	[90]

*Primary construct*	
Sexual relationships and sexuality	[33, 38, 42, 49, 60, 71, 75, 80–84]
Personal relationships	[16, 32, 34, 44, 46, 47, 67, 72, 74, 78, 79]
Recreation and sport	[36, 37, 40, 48, 61, 85, 86]
Social integration/participation	[35, 39, 52, 58, 66, 73, 88]
Technology	[45, 53, 56, 64, 87]
Bladder and bowels	[43, 54, 55, 57, 90]
Employment	[50, 51, 76, 89]
Lived experience	[18, 41]
Outcomes	[69, 70]
Quality of life	[63, 68]

*Methodology*	
* Qualitative*	
Phenomenology	[34, 36, 41, 42, 49, 57, 60, 61, 77, 81, 83, 87, 89]
Descriptive/exploratory	[33, 55, 64, 68, 80, 86]
Grounded theory	[17, 40, 59]
Narrative	[52, 73, 82]
Others/not stated	[16, 18, 50, 63, 65, 67, 74, 76, 84]
*Quantitative*	
Correlational/comparative	[32, 35, 37–39, 46–48, 53, 54, 58, 66, 69–72, 78, 79, 88, 90]
Intervention study	[44, 62]
Case control	[43]
Survey	[75]
Randomized controlled trial	[51]
*Mixed methods*	[45, 56, 85]

*Participants*	
*Gender*	
Women only	[36, 49, 56, 59, 60, 73–75, 80–82]
Men only	[33, 38, 42, 44, 77–79, 83]
Gender breakdown unclear	[39, 46, 58, 61, 64]
*Diagnostic groups/comparison groups*	
Mixed diagnostic groups	[38, 39, 55, 65] Remaining studies focused exclusively on SCI
Includes non-SCI control, comparison, or family group	[16, 41, 46, 75, 78, 79]

**Table 3:** Factors which facilitate or impede interpersonal interactions and relationships after SCI.

Impeding factors	Facilitating factors
*Body structure and function factors*	*Body structure and function factors*
Level of impairment [32, 42, 58, 71]	Decreased impairment [37]
Incontinence [42, 54, 55, 60, 75, 82]	Sildenafil [38]
Bowel dysfunction [54, 57]	
Poor health [58]	Good health [47, 63, 72]
*Activities factors*	*Activities factors*
Bladder management [55, 90]	Bladder management [43, 71]
Lack of independence [34, 59]	Independence [46, 83]
Need for preparation means no spontaneity [63, 81]	Manual wheelchair use (point of distinction) [33]
Time required for tasks [66]	
*Participation factors*	*Participation factors*
Unemployed [32, 47, 66]	Recreation and sport [34, 36, 37, 40, 61, 86]
	Working [50, 51, 76, 89]
	Social integration [47]
*Environment factors*	*Environment factors*
*Social environment factors*	*Social environment factors*
Institutional constraints [68, 80]	Clubs, businesses, and organizations [58, 66]
Social bias [18, 33, 41, 42, 63, 80, 81, 84, 88]	
Have not met the right person [75, 81]	Social rehabilitation [16, 17, 44, 52, 59, 68, 85]
Violent aetiology [32]	Vocational rehabilitation [51, 89]
*Resource factors*	*Resource factors*
Financial pressure [34, 37, 58, 82]	Assistive technology [66, 87]
No communication technology [53]	Communication technology [45, 53, 56, 64, 66, 81, 87]
Poor information [42, 49, 80, 81]	Equipment [58]
	Income [39, 66]
	Information and education [82, 88]
	Service dogs [62]
	Transportation [58, 71]
*Physical environment factors*	*Physical environment factors*
Access & terrain [52, 55, 58, 63, 66, 67, 82]	Less residential density and development [35]
Climatic conditions [66]	
*Supporter factors*	*Supporter factors*
Impact of caregiver duties [41, 82]	Being married [71]
Friends or partner could not handle it [18, 65, 67, 73, 82]	Raising children [34]
Life situation precludes intimacy [42, 71]	Instrumental support [79]
Preinjury relationship issues [49]	Reciprocity and mutuality [16, 17, 46, 52, 68, 77–79]
Pressure from family [82, 84]	Partner support [16, 34, 72, 80–82]
Passage of time associated with relationship breakdown [32]	Relationships started after injury [49]
	Strong preinjury relationship [16, 69]
	Quality time [34, 41]
	Social support [17, 58, 59, 66, 72, 78, 81, 88]
	Peer support [74, 81]
*Personal factors*	*Personal factors*
*Fixed factors*	*Fixed factors*
Age [32, 39, 42, 70]	Age [37, 47, 71]
Educational level [39]	Cognitive ability [37]
Ethnicity [37, 47]	Culture [16]
Gender [71]	Higher education [47, 53]
Anxiety & avoidance in attachment style [46]	Gender [39]
	Secure attachment style [46]
*Modifiable factors*	*Modifiable factors*
Bitterness after breakups [49]	Broadening personal definition of sexuality [42, 49, 80–83]
Decreased sexual interest [75]	Helpful cognitive schemas [79]
Social discomfort [59]	Faith [16]
Fear of rejection [82]	High social self-monitoring [48]
Poor self-image [42, 49, 59, 75, 80–82, 84]	Positive self-image [36, 42, 52, 81, 88]
Not discussing disability [81]	Adaptation over time [81]
Not proactive [33]	Accepting help [65]
Opting out [49]	Effort [77]
Lack of social skill [52]	Self-presentation [52, 59, 80–82]
	Proactive [52, 67, 74, 82]
	Communication [16, 49, 77, 80, 82]
	Valuing relationships and being valued [16, 34, 52, 68, 82]
	Patience and acceptance [16]
	Good interpersonal skills [33]
	Gaining sexual experience [42]
